# German version of the Chedoke McMaster arm and hand activity inventory (CAHAI-G): intra-rater reliability and responsiveness

**DOI:** 10.1186/s12955-020-01499-6

**Published:** 2020-07-23

**Authors:** Frank Behrendt, Julia Charlotte Rizza, Fabian Blum, Zorica Suica, Corina Schuster-Amft

**Affiliations:** 1 Research Department, Reha Rheinfelden, Rheinfelden, Switzerland; 2 Institute for Rehabilitation and Performance Technology, Bern University of Applied Sciences, Burgdorf, Switzerland; 3 Department of Sport, Exercise and Health, University of Basel, Basel, Switzerland

**Keywords:** German version Chedoke McMaster arm and hand activity inventory, Responsiveness, Intra-rater reliability

## Abstract

**Background:**

The English version of the Chedoke Arm and Hand Activity Inventory is a validated, upper-limb measure with the purpose of assessing functional recovery of the arm and hand after a stroke. A German translation and cross-cultural adaptation was recently produced and demonstrated high validity, inter-rater reliability and internal consistency. As a follow-up, the present study evaluated the intra-rater reliability and responsiveness of the CAHAI-G for the long and all shortened versions.

**Methods:**

The CAHAI-G and the Action Research Arm Test were assessed on three different measurement events: upon entry (ME1), two to 3 days after entry (ME2), and after three to 4 weeks (ME3). For the intra-rater reliability analysis, the ME1 CAHAI assessments were recorded on video and rated by three therapists to obtain the intraclass coefficients (ICC). The data of all three MEs were analysed in a group of stroke inpatients for the evaluation of responsiveness. To test for responsiveness, the CAHAI-G change data were compared to concurrent instruments: The Global Rating of Change-questionnaire and the Global Rating of Concept-questionnaire. Both served as external criteria. For all CAHAI-G versions (7, 8, 9 or 13 items), the same analysis procedures for the evaluation of the responsiveness parameter were performed.

**Results:**

In total, 27 patients (9 females, age 63 ± 13.7) were enrolled in the study. The ICCs for the intra-rater reliability were calculated to be between 0.988 and 0.998 for all CAHAI versions. Responsiveness parameters were as follows from CAHAI-G 7 to 13: Minimal Detectable Change (MDC_90)_ 5.3, 6.0, 6.1, 8.2; Pearson’s correlation coefficients CAHAI-Gs with ARAT 0.365, 0.409^*^, 0.500^**^, 0.597^**^. The Area und Under the Curve and the Minimal Clinical Important Difference values for all CAHAI-G versions and the three external criteria ranged between 0.483 to 0.603 and 2.5 to 9.0, respectively.

**Conclusion:**

In addition to the high validity, inter-rater reliability and internal consistency, the CAHAI-G revealed high intra-rater reliability. The data also suggest an adequate responsiveness of the CAHAI-G versions 9 and 13.

## Background

Regaining arm and hand function is one of the most frequently identified goals by patients after a stroke [1]. Upper limb disorders are present in 50–70 and 40% of persons with stroke in the acute and chronic phase respectively [2].. The lack of function in the paretic hand or arm directly affects the quality of life, and affected patients value any upper limb recovery [3, 4]. Impairments of the upper extremities after a stroke can be objectified by the use of specific assessments. For an improvement of the use of the paretic upper limb in the daily lives of stroke survivors, it is essential to have objectively-assessed outcome measures to set appropriate rehabilitation goals and to evaluate the treatment progress [5–7].

The Chedoke Arm and Hand Activity Inventory (CAHAI) can be used for this purpose as it is a validated objective assessment designed specifically for evaluating activities of daily living (ADL) of the affected upper extremity after a stroke [1]. The studies on the original, English language version of the CAHAI with 13 items and the shortened versions with 9, 8 or 7 items have shown good psychometric properties. Good reliability was found with high inter-rater reliability for all four versions [8], high test-retest reliability [9] and also high internal consistency [1]. The CAHAI was found a valid assessment, and the comparison of the CAHAI with both the Action Research Arm Test (ARAT) [10] and the Chedoke-McMaster Stroke Assessment [11] revealed an excellent convergent validity [8]. Additionally, evaluation of quality factors also proved the ability of the CAHAI to distinguish a patient with an improved condition from a patient with an unchanged condition [12].

Considering these positive characteristics of the English version, the CAHAI was culturally adapted and translated into several languages. The evaluation of certain psychometric properties of all short and the long German CAHAI (CAHAI-G) versions also showed a high inter-rater reliability and internal consistency [5]. Furthermore, the correlation between the CAHAI-G and CMSA subscales for hand and arm was moderate to strong reflecting a sufficient convergent validity [5]. Thus, the authors stated that CAHAI-G is a valid and reliable assessment of bilateral upper extremity performance in activities of daily living (ADL), and recommended its use in German-speaking stroke patients.

However, certain psychometric factors of CAHAI-G have not yet been investigated. Accordingly, in addition to the inter-rater reliability and the internal consistency tested before [5], the present study aimed to evaluate the responsiveness and intra-rater reliability of the German CAHAI versions. This would, on the one hand, provide certainty about the ability of the CAHAI-G to detect clinically important changes in the course of therapy in stroke patients with upper extremity paralysis. On the other hand, it would bring certainty about the reliability of the CAHAI-scores that are assessed at intervals of the same therapist which is common routine in everyday clinical practice. Since intra-rater reliability has not yet been investigated for the English version either, the study results could also provide hints about this psychometric property of the CAHAI in general in its application with stroke patients.

## Methods

### Study design and procedures

**Fig. 1:**
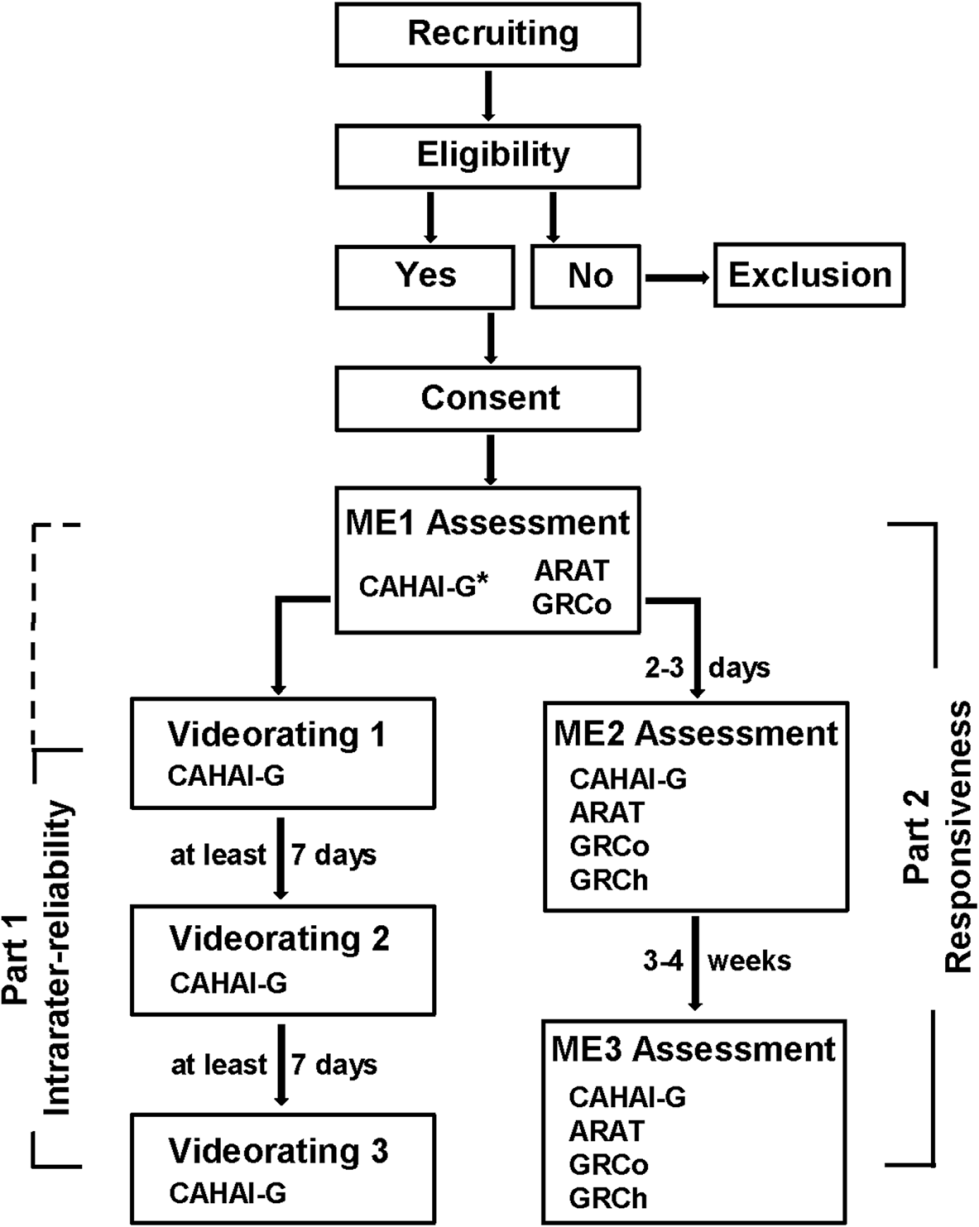
ARAT – ActionResearchArmTest; CAHAI–G - Chedoke Arm and Hand Inventory (German version); GRCh - Global Rating of Change, GRCo - Global Rating of Concept; ME1/2/3 – Measurement event 1/2/3; * Patients were recorded on video during CAHAI-G assessment at ME1; Please note that in some cases the first rating was performed during the ME1 assessment

The patient study was conducted in a rehabilitation centre in the German-speaking part of Switzerland. It consisted of two parts: Part 1 concerned the assessment of the intra-rater reliability and part 2 evaluated the responsiveness of the CAHAI-G. Figure 1 illustrates the study design. All procedures complied with the guidelines of good clinical practice and the Declaration of Helsinki. Ethical approval was obtained from the responsible Swiss ethics committee of Northwest and Central Switzerland EKNZ (reference number 2017–00161).

All new patients admitted to the clinics’ inpatient department between March and December 2017 were checked for inclusion and exclusion criteria. With this approach to patient recruitment, a rather heterogeneous patient cohort could be expected in terms of parameters such as time since stroke, age or severity of impairment. Given this likely heterogeneity, one could a priori deduce that patient change was also heterogeneous. During their stay the patients underwent an individual multimodal rehabilitation programme. This included physiotherapy and occupational therapy, psychotherapy, physical therapies, medical training therapy, music therapy, and speech and language therapy. All patients were screened on admission to the rehabilitation unit for possible compliance with inclusion and exclusion criteria (Table 1) via the clinics’ database. Prior to the first measurement event (ME1), patients were informed about the study in oral and written form and written informed consent was obtained from all participants. CAHAI-G and ARAT were administered by one or the other of two investigators in charge, both intensively trained in the administration of the assessments beforehand. The GRCo questionnaire was filled in by the patients themselves. For the evaluation of the responsiveness, two further consecutive measurement events were performed: two to 3 days after ME1, and again after three to 4 weeks. It was ensured that the assessments at the three MEs were conducted by the same investigator for every individual patient. At ME2 and ME3, both patients and treating therapists were asked to fill in the GRCh.

**Table 1:** Patient selection criteria

Inclusion criteria	Exclusion criteria
● Patients after first-ever stroke (ischaemic, haemorrhagic)	● Pre-existing functional impairment of the upper extremities
● ≥ 18 years	● Severe cognitive deficits
● able to sit on a normal chair without armrests	● Additional neurological or psychiatric disorders
● German-speaking	● Pain during the measurement

During the ME1 assessment each patient was recorded on video while she/he was tested with the CAHAI-G. The position and viewing angle of the video camera was identical for each recording throughout the study. Based on these videos, the intra-rater-reliability was independently evaluated by three therapists three times per therapist with at least 7 days [13] between two successive evaluations. The raters who completed the video ratings included two physiotherapists and a sports scientist. They could stop the video once per test item to watch the recording of the specific item a second time.

### Patient selection criteria and recruitment

Patients were eligible for study participation if they fulfilled the selection criteria listed in Table 1.

### Outcome measures

#### German version of the Chedoke arm and hand activity inventory

The CAHAI was developed to assess functional ability of the paretic arm and hand [1]. It is a performance test using functional items and is not designed to measure the client’s ability to complete the task using only their unaffected hand, but rather to encourage bilateral function. The original CAHAI consists of 13 functional tasks. There are shortened versions with 9, 8 and 7 items. In this study on the German version, the full 13-item version was administered, and the scores of the first 7, 8, and 9 items were then summed to derive participants’ scores on the shortened versions. All four translated German versions of the CAHAI were recommended as reliable and valid performance-based measures to assess bilateral upper limb ADL task performance in clinical practice [5]. A high inter-rater reliability was calculated with ICCs for all four CAHAI-G versions ranging between *r* = 0.96 and *r* = 0.99 (*p* < 0.001). Correlation between the CAHAI-G and CMSA subscales for hand and arm was *r* = 0.74 (*p* < 0.001) and *r* = 0.67 (*p* < 0.001) respectively. Internal consistency of the CAHAI-G versions ranged between α = 0.974 and α = 0.979 [5].

#### ActionResearchArmTest

The ARAT is a commonly used standardized and reliable assessment for stroke rehabilitation. It is a proven standardized evaluative measure to evaluate specific changes in upper limb function among individuals who sustained cortical damage resulting in hemiplegia. The ARAT, as well as the CAHAI, requires an examiner to transform observations of a patient’s performance into a score to set treatment goals and select appropriate treatment methods. Items comprising the ARAT are categorized into four different subscales (grasp, grip, pinch and gross movement) and arranged in order of decreasing difficulty, with the most difficult task examined first, followed by the least difficult task. The task performance is rated by the examiner on a 4-point scale, ranging from 0 (no movement at all) to 3 (movement performed normally). The ARAT revealed high values of test-retest reliability (ICC = 0.965, *r* = 0.68), interrater-reliability (ICC = 0.997, *r* = 0.999) and construct validity comparing it to the Box and Block Test (0.951) and to the Fugl-Meyer Assessment (0.925) [14].

#### Global rating of change / of concept

The questionnaires Global Rating of Change [15] and the Global Rating of Concept [16] both served as external criteria in this study. The Global Rating of Change was used to investigate the extent to which the change measured by the CAHAI was actually perceived by the patient as change. For this, the patients themselves estimated the change in the function of the upper extremity based on a 7-level scale. They were asked the following question: “How do you now assess the function of your stroke-affected arm compared to admission to rehabilitation?” In addition to the patient-based Global Rating of Change, an assessment of the change in the patient’s upper limb function was obtained from the treating physiotherapist [17]. The following question was asked: “Compared to entering rehabilitation, how do you estimate the hand and arm function of the patient today?”

The Global Rating of Concept is an alternative for the Global Rating of Change and served as a further external criterion used to examine whether there is a change in the patient’s condition that is perceptible to the patient. The patients hereby estimated the activity of the affected upper extremities in the last 7 days. For this purpose, following Nixon et al. [18], it was asked: “In the last seven days, how hard has it been for you to cope with everyday tasks (such as dressing, grooming)?” This was answered based on the following 5 point scale of responses: (5) not difficult at all, (4) somewhat difficult, (3) rather difficult, (2) very difficult, (1) not possible at all. The possible responses were taken from the subscale for the manual function of the Stroke Impact Scale [19].

### Data analysis

#### Part 1: intra-rater-reliability

Intraclass correlation coefficient (ICC) calculation was used to test for the intra-rater-reliability. The ICC (2,1) type *two-way random-effects*, *single rater/measurement*, *absolute agreement* was chosen [20, 21]. It is suitable for determining intra-rater reliability of repeated assessments of the same measurement by a single rater [22] and was individually applied for each rater. The use of a random-effects model means that the study results on the reliability of the CAHAI-G are generalizable. According to the recommendations of Portney and Watkins (2015), an ICC of 0.800 suggests good and an ICC of > 0.900 very good reliability [23].

#### Part 2: responsiveness

The term *responsiveness* was defined as the ability of an instrument to measure a meaningful or clinically important change in a clinical state [24]. Responsiveness has become a critical criterion for the selection of outcomes measures in studies of treatment effectiveness. There is a clear distinction between *responsiveness* and *sensitivity to change* which is defined as the ability of an instrument to measure change in a state regardless of whether it is relevant or meaningful to the decision-maker [24]. Thus, in order to get clinically significant information we decided to evaluate responsiveness. The methods used in this study have been selected from various recommendations for assessing responsiveness [17, 25, 26] as it is known that there is no single characteristic or method that is considered the gold standard [27]. It was suggested that the targeted selection of appropriate change coefficients should be based on the specific change characteristics of a sample [28]. Stratford and Riddle [28] reported that depending on whether the actual change in the sample is likely to be homogeneous or heterogeneous, an appropriate selection of coefficients of change should be made.

#### Minimal detectable change

The MDC describes the smallest measurable change that is no longer due to a measurement error or random fluctuations. If a measured change value exceeds the MDC, it can be assumed that it is neither a random fluctuation nor a measurement error [29]. In the literature, the MDC of a measure was considered satisfactory when the MDC was less than 10% of the highest possible score on the measure [30, 31]. An MDC with a confidence interval of 90% indicates how high the number of random changes can be in 90% of the stable patients after repeated test procedures. A confidence interval of 90% (MDC_90_) was already chosen in previous studies for the evaluation of responsiveness of the original CAHAI [8, 12]. The MDC_90_ is based upon the standard error of measurement (SEM) of the change: *MDC*_*90*_ *= SEM × 1.65 x*$\sqrt{2}$ [32].

The SEM was calculated as *SEM = s x*$\sqrt{1-r}$, where s represents the standard deviation of the values collected at ME1 while r stands for the test-retest reliability. The test-retest reliability was determined on the data collected at ME1 and ME2 by calculating the ICC. For this purpose, the model *two-way mixed-effects* and the type *absolute agreement* was applied [22].

#### Correlation CAHAI-G / ARAT

CAHAI-G change scores were compared to the ARAT change scores. Pearson’s correlation coefficients between change scores from ME1 to ME3 of all four CAHAI-G versions and the ARAT were calculated to evaluate whether the instruments respond similarly [33].

#### Area under receiver operating characteristic (ROC) curves

Additionally, the *Area under Curve* (AUC) was also determined as a characteristic of responsiveness of the CAHAI-G. With the help of this information, a cut-off value for the minimum clinically significant change could be determined. On the basis of the AUC, it was also evaluated how well the CAHAI-G can distinguish between a clinically relevant change and no change. To assign the corresponding patient data to the ROC analyses (A-D), both global ratings of change were used as external criteria. An increase of one point was determined as the criterion for selection. This cut-off was chosen as it was planned to include patients with even the smallest improvement. A ROC curve was created for each of the four categories. A) The selection for this category was made on the basis of the patients’ own assessments. Only patients who assessed the functionality of their affected upper limb as improved based on the Global Rating of Change were included in the analysis here. B) The therapists’ assessment based on the Global Rating of Change was used for this category. All patients who were classified as improved by the therapist were selected for the analysis. C) The classification for this category was based on the Global Rating of Concept from ME1 to ME3. Again, only the improved patients were included. D) In addition, a fourth ROC curve was generated, taking into account only those data where the patient’s assessment was consistent with the therapist’s assessment [17]. Here it was considered an agreement between patient and therapist if the direction of the judgment was the same meaning that the level of improvements was not taken into account. It could thus be that the data of one patient were included in different analyses.

The different ROC curves were also used to obtain four corresponding *Minimal Clinically Important Difference* (MCID) values. The MCID represents a cut-off value above which a measured change can be interpreted as clinically relevant [26] and refers to the point nearest to the upper left-hand corner that jointly maximises sensitivity and specificity.

## Results

### Patient descriptives

A convenience sample of 32 consecutive patients was enrolled during the recruiting period. Five dropouts were recorded for instance due to early discharge from the clinic leaving a dataset of 27 patients (Table 2) for analysis (9 females, mean age 63 ± 13.7). Only datasets of patients without missing data from the three measurement events were included in the analysis. For the recruited patients, the time since stroke (17 ischemic, 10 haemorrhagic) ranged from 13 to 82 days, mean time was 27 days. Mean number of days between ME1 and ME2 was 2.6 (±2.3) days and 16.7 (±6.3) days from ME1 to ME3. Mean National Institutes of Health Stroke Scale (NIHSS) - score was 7.27 (SD 5.01) and EBI (Extended Barthel Index) - score was 49.8 (SD 10.7) on average.

**Table 2:** Scores of all outcome measures

	Mean	SD
**ARAT ME1**	38.5	17.6
**ARAT ME2**	40.9	15.6
**ARAT ME3**	41.8	17.0
**CAHAI-G ME1**	65.8	23.7
**CAHAI-G ME2**	69.8	24.0
**CAHAI-G ME3**	73.0	23.6
**GRCo_ME1**	3.87	1.07
**GRCo_ME2**	4.24	0.86
**GRCo_ME3**	4.46	0.68
**GRCh_P_ME2**	1.52	1.10
**GRCh_P_ME3**	1.71	1.19
**GRCh_Th_ME2**	1.18	1.10
**GRCh_Th_ME3**	1.41	1.16

Legend: *ARAT* ActionResearchArmTest (max. Score 57), *CAHAI-G* Chedoke Arm and Hand Inventory - German version (max. Score 100), *GRCo* Global Rating of Concept (max/min. Score 5/1), *GRCh_P/Th* Global Rating of Change by patient / therapist (max./min. Score 3/− 3), *ME* Measurement Event.

The included patients formed a rather heterogeneous cohort with regard to the level of arm function as there were patients with minimal but also with almost completely preserved arm function. However, on average, the ARAT and CAHAI G values showed a moderate impairment of arm function, which overall represents the usual patient spectrum.

### Intra-rater-reliability

The mean ICC scores for every CAHAI-G version are provided in Table 3. The scores revealed very good intra-rater reliability of > 0.900 throughout the versions with slightly rising ICC values from the 7-item version to the 13-item version.

**Table 3:** ICCs and measures of responsiveness for all four CAHAI-G versions

		CAHAI-G 7	CAHAI-G 8	CAHAI-G 9	CAHAI-G 13
**ICC (2,1)**		0.991	0.991	0.992	0.993
**CI**		0.984–0.996	0.984–0.995	0.985–0.996	0.988–0.997
**MDC**_**90**_		5.3	6.0	6.1	8.2
**Pearson’s correlation coefficients**		0.365	0.409^a^	0.500^b^	0.597^b^
**AUC/MCID**	*A) GRCh-P*	0.603 / 4.5	0.536 / 2.5	0.571 / 2.5	0.599 / 2.5
	*B) GRCh-T*	0.510 / 3.0	0.483 / 3.5	0.521 / 5.5	0.590 / 5.0
	*C) GRCo*	0.543 / 3.5	0.526 / 3.5	0.531 / 4.0	0.554 / 9.0

Legend: *ICC* intraclass correlation coefficient, *CI* confidence interval (95% confidence level), *MDC*_*90*_ Minimal Detectable Change with a confidence interval of 90%, *AUC* Area Under Curve, *MCID* Minimal Clinical Important Difference, *Global Rating of Change-P* Global Rating of Change – rated by the patients, *Global Rating of Change-T* Global Rating of Change-rated by the therapists, *GRCo* Global Rating of Contrast (rated by the patients), ^a^correlation is significant at the 0.05 level (2-tailed), ^b^correlation is significant at the 0.01 level (2-tailed.

### Responsiveness

As shown in Table 3, the MDC_90_ values increased from the CAHAI-G 7-item version to the 13-item version from 5.3 to 8.2. This gives a good indication of how well small changes can be measured with the different CAHAI-G versions. Pearson’s r-values for CAHAI-G increased with the number of rated CAHAI tasks with no statistical significance for the 7-item CAHAI-G version. Only versions 9 and 13 revealed a strong correlation, while the 8-item version only showed a moderate correlation. On this basis, one can assume that at least versions 9 and 13 measure the same as the ARAT. With regard to the AUC, none of the values exceeded 0.7. Thus, all CAHAI-G versions could not distinguish well enough between a clinically relevant change and no change. Across the three external criteria and four different CAHAI-G versions, the MCID values ranged between 2.5 and 9.0.

Table 4 gives an overview of the sample distribution according to the selected cut-off of one point for the three external criteria and according to the CAHAI-G change scores based on the 13-item version using the corresponding MCID values.

**Table 4:** Confusion matrices for the three different categories / external criteria and the CAHAI-G (13 items) change scores

CAHAI-G		Category A GRCh-Patient		Category B GRCh-Therapist		Category C GRCo	
		improved	not imp.	improved	not imp.	improved	not imp.
	improved	6	14	6	7	6	4
	not imp.	1	6	2	12	8	9

Note that to distinguish between ‘improved’ and ‘not improved’ according to the CAHAI-G change scores, a different MCID value was used for each category (see Table 3)

## Discussion

The aim of the present study was the evaluation of the intra-rater reliability and responsiveness of the German CAHAI versions 7, 8, 9 and 13. For this purpose, we included a sample of stroke patients in the study and examined them based on video ratings for intra-rater reliability or repeatedly at three specific time points for different measures of responsiveness. We found that all CAHAI-G versions have a very good intra-rater reliability. Versions 9 and 13 revealed a good responsiveness based on the results of the correlation analysis. Our results may provide an indication for the applicability of the different CAHAI versions in German-speaking patients.

### Intra-rater reliability

The intra-rater reliability is of interest to all clinicians interested in the reproducibility of their measurements with regard to the assessments they use. Therefore, the analysis of the corresponding ICC values was an important goal of this study. The ICCs of all evaluated CAHAI-G versions showed strong correlations, indicating excellent reproducibility and intra-rater reliability. However, a comparative classification of the presented ICC values with regard to other language versions is not possible, since this quality factor was investigated for the first time for the CAHAI. Nevertheless, the ICCs of the different CAHAI-G versions are indeed acceptable for clinical measures [34] and suggest its application with patients.

### Responsiveness

High responsiveness is important for any measurement tool designed to evaluate meaningful change [35]. Values representing the MDC are useful for clinicians in determining whether an individual patient has achieved real changes [36]. In the literature, an MDC’s share of the overall score of less than 10% was considered satisfactory [31]. In a comparison of the psychometric properties of four clinical measures (upper-extremity subscale of the Fugl-Meyer Assessment, upper-extremity subscale of the Stroke Rehabilitation Assessment of Movement, ARAT, Wolf Motor Function Test) only the ARAT and the upper-extremity subscale of the Fugl-Meyer Assessment were below 10% of their corresponding highest scores [31]. The MDC_90_ scores of CAHAI-G 7–13 represent percentages of its total scores of 10.8, 10.7, 9.7 and 9.0 suggesting good responsiveness of CAHAI-G 9 and 13. Thus, changes of more than 6.1 and 8.2 points for the latter two versions are not likely to be attributable to chance variation or measurement error and can be interpreted by clinicians as a real change with 90% confidence. The MDC values are about two CAHAI points larger than the original CAHAI’s MDC, which might be considered similar.

Based on Barreca et al. [8] we assessed responsiveness by correlating the CAHAI-G’s scores with the ARAT scores [8] which was recently recommended as the measurement standard for the assessment of upper limb function [37]. A correlation analysis was recommended in cases where the sample is a single heterogeneous group of patients with varying degrees of change [28], which applies to the present study sample. CAHAI-G 9 and 13 revealed a strong positive relationship with the ARAT. This level of correlation is indeed lower as compared to the correlation values obtained between the CAHAI and the ARAT scores (*r* = 0.86) of the English CAHAI versions by Barreca and colleagues [8]. An underlying cause might be that slightly different scoring methods of the ARAT were used. A manual that provided a detailed, standardized approach to scoring the ARAT was only published in 2007 [38]. This had become necessary as the lack of a unified approach led to an unacceptably high intersite variance. However, when considering the correlation coefficients alone, the application of CAHAI-G 9 or 13 should be preferred accordingly.

The area under the ROC curve (AUC) describes the probability with which the CAHAI correctly distinguishes between patients with improved and unchanged upper extremity function. The AUC values in this study ranged between 0.48 and 0.60, depending on the external criterion, which is clearly below the required minimum value of 0.70 proposed by Terwee et al. [39]. The probability of correctly differentiating between patients with improved and unchanged upper extremity function was hence higher with an AUC value of 0.86 [12] for the English CAHAI than for all the different German CAHAI versions. A possible reason for the AUC results could be the overall small sample size, which in some cases only allowed a small sample size for the cells of the 2 × 2 matrices in Table 4. Another reason to consider is also the sampling method used. Barrerca et al. (2006) adopted a stratified sampling of patients with stroke of different chronicity and upper limb impairments [12]. A more equal distribution of participants could have been achieved by adopting a similar sampling method. As there are cells that have less than 5 participants (Table 4) this would have affected the ROC analysis and may explain the low AUC and MCID values. In addition, the selection of only at least 1 point change as cut-off corresponded only to a slight change, if any, measured with the CAHAI-G. Further, it is important to note that Barreca et al. (2006) used a different study design to assess the ability of the CAHAI to measure change rather than an external criterion to rate their sample [12]. They rated their sample of stroke patients in terms of the severity of impairment and also post-stroke time. It was expected that patients with a severe impairment, whose stroke was three to 12 months ago, would change less than patients with mild to moderate impairment, whose stroke was less than 8 weeks ago. The aim was to have cohort groups with a more homogeneous amount of change. These methodological differences could be part of the explanation for the discrepancy.

The ROCs were also analysed to determine the minimal clinically important differences the patients or therapists would identify as important. In the present study, three MCID values were generated from a different perspective of change: A) from the patient’s perspective, based on the patient-based Global Rating of Change; B) from the therapist’s perspective using the patient-based Global Rating of Change and C) the change in coping with everyday activities based on the Global Rating of Concept. In order for the MCID to provide reliable interpretations of clinical significance, the MCID must exceed the MDC. In this way, it can be ruled out that random fluctuations or measurement errors are erroneously interpreted as clinically relevant changes. We could find MCID values that exceed those of the MDC for the CAHAI-G 9 in combination with the Global Rating of Change-P and also for the CAHAI-G 13 in combination with the Global Rating of Concept. All other MCID values for versions CAHAI-G 7 and 8 did not allow for a reliable interpretation of a clinically relevant change. Possible reasons that MCID did not exceed MDC could be that on the one hand, the cut-off of only one point may have led to lower MCID values, as they would probably have been higher if the predetermined difference between “improved” and “not improved” based on the external criteria had been more than just one point. On the other hand, the positive correlation between the change scores in the external criteria and the CAHAI-G change scores was not as strong as assumed. This could also have influenced the MCID values.

### Strengths and limitations

We conducted a comprehensive assessment of the responsiveness of the CAHAI-G, including some key parameters that have not yet been studied for the original English version. A limitation is certainly that we did not conduct a pilot study to determine the likely change characteristics of our patient population, which would have been the best approach. The importance of specifying the change characteristic in advance is that it helps to choose the appropriate change coefficient for the analysis of responsiveness, or at least the family from which the change coefficient should be selected. Concerning the values of change in this population, it must be noted that the greatest attention should be paid to correlation analysis. The relevant results imply the use of CAHAI-G versions 9 and 13. However, some results of responsiveness in this study were inferior in comparison to the original CAHAI. Possible reasons have already been mentioned in the discussion and certainly highlight that a study with more rigorous design is needed to re-evaluate the responsiveness of the CAHAI-G rather that it is not responsive.

Another limitation might be the fact that the recognition of fine motor movements on the basis of video recordings was perhaps a bit more difficult than the assessments during the actual execution leading to minor differences in the ratings. However, video-ratings to determine intra-rater reliability are recognized as a standard procedure which resulted in good intra-rater reliability scores for different assessments [40–43]. A further factor influencing the results might have been the differences in the rating experience. Two of the raters had several years’ experience as treating therapists and also in using the CAHAI-G at the time. The third rater was a recent graduate in sports science who had received extensive training for 3 months in advance by the two other raters and had worked with patients at the rehabilitation center already before his graduation. All three showed comparable results, suggesting that the assessment is also applicable to raters with less experience in stroke rehabilitation.

## Conclusions

Numerous outcome measures evaluating motor function of the upper extremity have been developed of which the ARAT, the Fugl-Meyer Test-arm subscale, and the CAHAI have been used and cited most frequently. As the CAHAI was developed to overcome certain shortcomings of other measures by reflecting everyday-activities it seemed worthwhile to develop a translated and adapted version of the CAHAI for the application with German-speaking patients. The different quality factors of the CAHAI-G concerning the validity, inter-rater reliability and internal consistency which have been subsequently evaluated after its translation and adaption proposed its usage as a valid and reliable assessment for bilateral upper limb performance in ADL. The high level of intra-rater reliability found in the current study strongly supports this recommendation. The evaluation of responsiveness suggests the use of the full CAHAI-G version or the 9-item version. Both are reliable and valid and revealed a rather acceptable responsiveness.

## Data Availability

The datasets used and/or analysed during the study are available from the corresponding author on reasonable request.

## Abbreviations

ADL: Activities of daily living
ARAT: Action Research Arm Test
AUC: Area under the curve
CAHAI: Chedoke Arm and Hand Activity Inventory
CI: Confidence interval
Global Rating of Change-P: Global rating of change – rated by the patients
Global Rating of Change-T: Global rating of change – rated by the therapists
ICC: Intraclass correlation coefficient
MCID: Minimal clinical important difference
MDC: Minimal detectable change
ME: Measurement event
ROC: Receiver operating characteristics
SEM: Standard error of measurement
SRM: Standardized response mean
